# A Tuneable, Photocurable, Poly(Caprolactone)-Based Resin for Tissue Engineering—Synthesis, Characterisation and Use in Stereolithography

**DOI:** 10.3390/molecules26051199

**Published:** 2021-02-24

**Authors:** Jonathan Field, John W. Haycock, Fiona M. Boissonade, Frederik Claeyssens

**Affiliations:** 1 The School of Clinical Dentistry, The University of Sheffield, Sheffield S10 2TA, UK; j.field@sheffield.ac.uk (J.F.); f.boissonade@sheffield.ac.uk (F.M.B.); 2 The Department of Materials Science and Engineering, The University of Sheffield, Sheffield S3 7HQ, UK; j.w.haycock@sheffield.ac.uk; 3 The Neuroscience Institute, The University of Sheffield, Sheffield S10 2HQ, UK

**Keywords:** microstereolithography, UV curable, polycaprolactone, PCL, methacrylate, methacrylation

## Abstract

Stereolithography is a useful additive manufacturing technique for the production of scaffolds for tissue engineering. Here we present a tuneable, easy-to-manufacture, photocurable resin for use in stereolithography, based on the widely used biomaterial, poly(caprolactone) (PCL). PCL triol was methacrylated to varying degrees and mixed with photoinitiator to produce a photocurable prepolymer resin, which cured under UV light to produce a cytocompatible material. This study demonstrates that poly(caprolactone) methacrylate (PCLMA) can be produced with a range of mechanical properties and degradation rates. By increasing the degree of methacrylation (DM) of the prepolymer, the Young’s modulus of the crosslinked PCLMA could be varied from 0.12–3.51 MPa. The accelerated degradation rate was also reduced from complete degradation in 17 days to non-significant degradation in 21 days. The additive manufacturing capabilities of the resin were demonstrated by the production of a variety of different 3D structures using micro-stereolithography. Here, β-carotene was used as a novel, cytocompatible photoabsorber and enabled the production of complex geometries by giving control over cure depth. The PCLMA presented here offers an attractive, tuneable biomaterial for the production of tissue engineering scaffolds for a wide range of applications.

## 1. Introduction

There is a need for tuneable, degradable materials for the production of tissue engineering scaffolds. Additive manufacturing, or 3D printing, is widely used in the field of tissue engineering for the production of 3D scaffolds and allows for the production of complex geometries, difficult to produce by conventional methods. Other advantages include the rapid prototyping capabilities allowing for an iterative design process, and the ability to utilise medical imaging data for the production of patient-specific implants [1,2]. Different forms of 3D printing exist, such as fused filament fabrication (FFF), selective laser sintering (SLS), and inkjet printing, all of which have applications in the fabrication of medical implants and tissue engineering scaffolds [3]. Stereolithography (SL), the oldest form of 3D printing, produces 3D constructs with the use of a laser (or other forms of UV/visible radiation) to cure successive layers of a photocurable, liquid resin. Since the invention of SL in 1984 [4], this technology has been developed to allow the production of high resolution, additive manufactured structures with around 20 μm resolution on commercially available stereolithography set-ups. The production of high-resolution structures is studied in microstereolithography (µSL), and these techniques have been used in tissue engineering for a wide variety of applications, such as the production of nerve guidance conduits (NGCs) [5,6,7], scaffolds for bone with multiscale porosity [8], cell-encapsulating hydrogels [9] as well as templates for electrospun corneal scaffolds [10]. Fabrication of structures with sub-micrometer resolution has also been made possible with the advance of 2 photon polymerisation (2PP), and this has been demonstrated with a range of degradable materials, including a photocurable poly(caprolactone)-trimethylene carbonate copolymer [11], poly(lactic acid) (PLA) [12], poly(glycerol sebacate) (PGS) [13] and most recently a urethane-based poly(caprolactone) [14]. This is of particular interest for in vitro cell culture and the study of cell-response to substrate micro-topography. For example, the influence of different sizes and shapes of features, in the order of 1 µm and below, has been studied on a variety of cell types [15,16,17,18].

Materials for stereolithography must be in the form of a photocurable prepolymer resin which solidifies/crosslinks under UV irradiation. To render a polymer photocurable, it must be functionalised by the addition of certain functional groups which facilitate crosslinking between prepolymer molecules. Acrylate or methacrylate groups are often added for this purpose due to their high reactivity [19]. Photocurable poly(ethylene glycol) (PEG) is widely used in SL, due to its commercially availability in dimethacrylated or diacrylated forms, and can either be used in its bulk form [20,21] or mixed with water and cured to produce a hydrogel [9,22,23]. Other photocurable hydrogels have been produced via this route, based on natural materials, including hyaluronic acid [24], gelatin [25,26], chondroitin sulphate [27], and alginate [28].

Recent developments have been made in the production of alternative photocurable, biodegradable materials for use in µSL and tissue engineering. Examples include poly(propylene fumarate) [29,30], poly(caprolactone fumarate) [31,32] and vinyl-terminated poly(caprolactone) [33]; however, these materials need reactive diluents to increase the reaction rate because of the relatively low reactivity of fumarate and vinyl functional groups. Caprolactone/trimethylene carbonate (CL/TMC) copolymers have also been made photocurable [11,34,35,36]. Coumarin functionalisation led to slow cross-linking and limited photocurability [34]; however, acrylated and methacrylated forms of CL/TMC copolymers have demonstrated a higher reactivity and have been used in SL [35,36] and 2PP [11], respectively. Other biodegradable polymers developed for stereolithography include a methacrylated poly(d,l-lactide) synthesised by Melchels et al. [37]. This was also co-polymerised with ε-caprolactone to produce a more flexible variant and used to produce 3D structures via µSL [38]. Additionally, polyglycerol sebacate methacrylate has been studied as a resin for µSL [6,13].

As demonstrated by its use in the various copolymers in the aforementioned studies, poly(caprolactone) (PCL) is well suited to use in the field of biomaterials and tissue engineering. It is biodegradable via hydrolysis [39,40] as well as via enzymatic action by lipases [41,42] and has been used in FDA approved medical devices (e.g., Monacryl sutures [43] and Capronor™, subdermal contraceptive implant [44]). PCL has been used in a number of tissue engineering applications such as biodegradable particles for drug delivery [45], knitted scaffolds for aortic valve [46] and electrospun scaffolds for various applications in bone [47], cartilage [48] and nerve [49]. In 3D printing, PCL’s low melting point of 60 °C makes it suitable for use in FFF and is widely used to produce woodpile scaffolds such as those by Hutmacher et al. [50]. Porous PCL scaffolds have also been produced via SLS for applications such as bone and cardiac tissue engineering [51,52,53].

There has been some research into the use of acrylated and methacrylated forms of PCL, such as the early work by Storey et al. involving the synthesis and thermal curing of PCL triol-methacrylate [54]. More recent work has been carried out by Elomaa et al. who produced a photocurable, methacrylated PCL resin and demonstrated cytocompatibility and 3D structuring capabilities with a commercial SL set-up [55]. Green et al. similarly demonstrated the use of acrylate-functionalised PCL [56].

Material properties such as Young’s modulus and degradation rate are important for tissue engineering scaffolds and materials should be selected or tuned to match the requirements for specific applications. Little work has been done to vary the mechanical and degradation properties of photocurable PCL. Approaches are usually limited to blending with different materials [57] or altering prepolymer molecular weight, as documented by Elomaa et al. and Green et al. [55,56], which typically results in small changes to the material properties.

The use of photocurable materials allows for additional control of polymer properties by controlling the degree of functionalisation, i.e., the degree of methacrylation (DM), of the prepolymer molecules. This affects the level of crosslinking which occurs during the photocuring process and in turn the crosslinking density in the cured material. By altering the crosslinking density, properties such as Young’s modulus and degradation rate can be modulated. The variation of polymer DM is a well-known practice and can be achieved by controlling various parameters in the chemical reaction, such as the reagent concentration, reaction time and reaction temperature. The relationship between increased DM and increased Young’s modulus or decreased degradation rate has been demonstrated with a wide range of natural polymers [24,25,26,27,28], as well as poly(glycerol sebacate) [13].

Another important consideration in the use of photocurable materials is the addition of photoabsorbers to control cure depth during stereolithography and allow for high resolution 3D structuring. A variety of photoabsorbers have been reported in literature but often with limited evidence of their biocompatibility. Examples include tinuvin [58], which has been shown to have possible cardiotoxic effects [59,60], and paprika extract [61], which may contain capsaicin, which is well known for its sensory nerve activation and inflammatory properties [62,63]. Recently, Grigoryan et al. demonstrated the use of tartrazine (a synthetic food dye), curcumin and anthocyanin (derived from turmeric and blueberries, respectively) as biocompatible photoabsorbers [64]. Here we consider β-carotene, as an alternative biocompatible photoabsorber. β-carotene is commonly found in foods and nutritional supplements and has been shown to exhibit beneficial anti-oxidant and anti-inflammatory properties in certain applications [65,66,67]. β-carotene absorbs highly within the violet-green range, commonly used in stereolithography, making it a suitable choice as a photoabsorber.

This paper investigates the synthesis of a photocurable PCL-methacrylate (PCLMA) prepolymer with varying degrees of methacrylation. We use a commercially available PCL prepolymer and vary the properties solely by changing the degree of methacrylation. The resulting material, with a range of mechanical properties and degradation rate, was used to produce a tuneable resin for use in laser-based 3D printing. The cytocompatibility of the PCLMA material was demonstrated, along with the ability to create complex 3D structures via stereolithography using β-carotene as a photoabsorber.

## 2. Results and Discussion

### 2.1. PCL Characterisation (NMR)—Effect of Reaction Conditions on the Degree of Methacrylation

**Figure 1:**
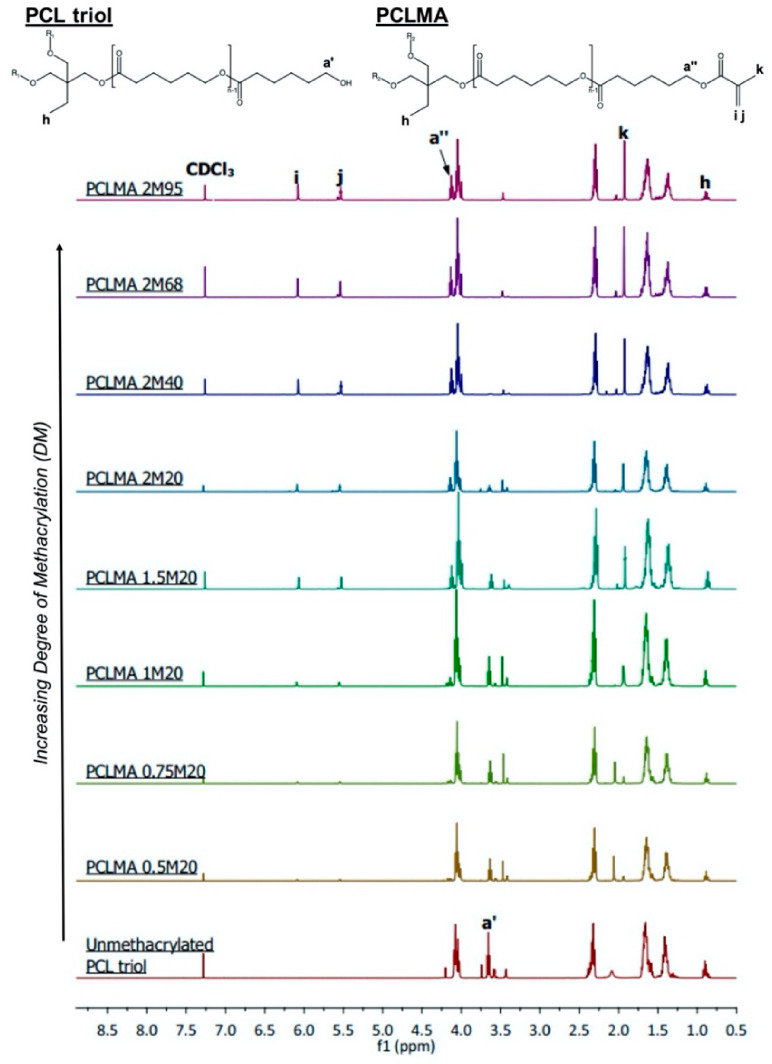
Proton NMR spectra of unmethacrylated and methacrylated PCL triol. The PCL triol and PCLMA molecule diagrams are displayed (top) with the hydrogen environments labelled (a′, a′′ and h-k) corresponding to the relevant peaks in the NMR spectra used to determine degree of methacrylation. a′′, i, j and k are present only in methacrylated PCL, so the size of these peaks increases with increasing DM.

PCL triol was methacrylated to varying degrees via reaction with methacrylic anhydride (MAA) and triethylamine (TEA) to produce photocurable poly(caprolactone)-methacrylate (PCLMA). Unmethacrylated PCL triol and PCLMA prepolymer samples were analysed by proton nuclear magnetic resonance (NMR) spectroscopy. Figure 1 shows example spectra of PCL triol and PCLMA with varying degrees of methacrylation and the molecule structures labelled with the corresponding hydrogen environments. Methacrylation was confirmed by the appearance of peaks i (6.09 ppm), j (5.55 ppm), k (1.93 ppm) and a′′ (4.14 ppm) which increased in size with increasing DM. The decrease in size of the a′ peak (3.64 ppm) indicates that the OH group from the original PCL triol molecule had become less prevalent in the reaction products, showing that these OH groups had been substituted for methacrylate groups.

**Figure 2:**
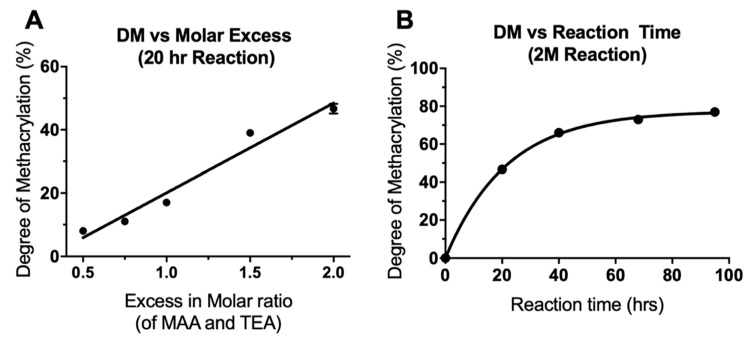
Alteration of the degree of methacrylation by (**A**) varying the molar ratio of reactants (linear regression curve fitted, R^2^ = 0.96) and (**B**) varying the reaction time (one phase decay curve fitted, R^2^ = 0.99). N = 1, *n* = 1, except for 2M20 where N = 3, *n* = 1 so mean ± SD shown only for 2M20 (error bars are too small to be observed in **B**).

Quantitative analysis was performed using the integrals of the i, j and a′′ peaks with the h peak (0.88 ppm) as a reference, equal to three protons. DM was seen to increase both with increasing molar excess of MAA/TEA and with increasing reaction time. This data is presented in Table 1, which also outlines the different reaction details and the notation used to name each reaction (see Materials & Methods, Section 3.1, for more information). As seen in Figure 2A, increasing the amount of TEA and MAA in a 20 h reaction, from a 0.5× molar insufficiency (0.5 M) to a 2× molar excess (2 M), resulted in a linear increase in the DM from 8% to 47%. Even with a molar excess of MAA and TEA, the methacrylation was not 100% efficient; however, increasing the reaction time of a 2 M reaction further increased DM. 40, 68 and 95 h reactions were carried out, resulting in DM of 66%, 73%, and 77%, respectively (Figure 2B). With longer reaction times, DM did not increase linearly but increased along an exponential plateau curve. As reaction time was increased, the rate of increase of DM dramatically slowed and levelled off at around 77%. This plateauing effect has been seen previously in the methacrylation of chondroitin sulphate and is due to an equilibrium state being reached between the reacting molecules [27]. The linear increase of DM with increasing molar excess of MAA and TEA suggests that a further increase in molar excess could be used if a DM of greater than 77% was required. Molar excesses of 20–100× have been previously used for methacrylation of gelatin and hyaluronic acid [24,25,26].

### 2.2. Effect of Degree of Methacrylation on PCLMA Mechanical Properties

**Figure 3:**
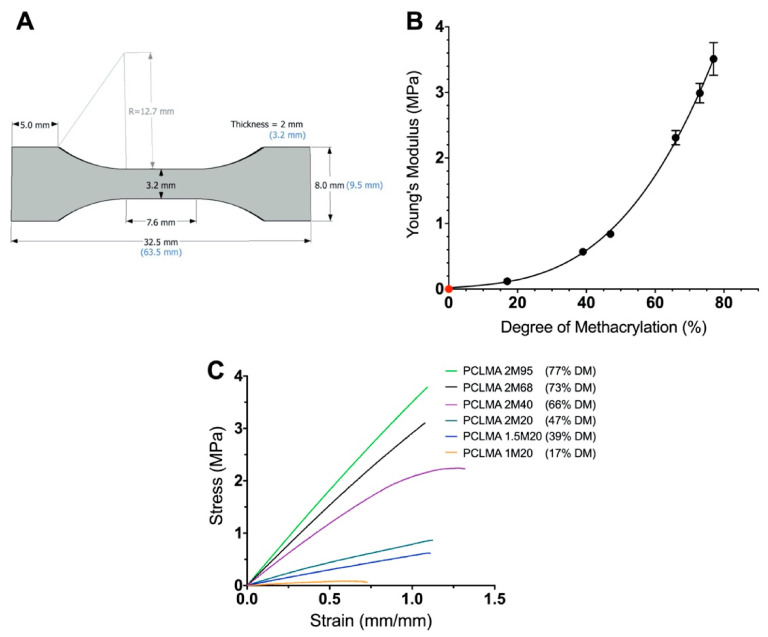
The effect of the degree of methacrylation on the Young’s modulus of the cured PCLMA. (**A**) specifications of the tensile test piece adapted from the ASTM D638-14 ‘Standard Test Method for Tensile Properties of Plastics’ Type V specimen; (**B**) the Young’s modulus of cured PCLMA plotted against the degree of methacrylation of the prepolymer. Values displayed mean ± SD, N = 1, *n* = 4–7. (N.B. The SD in the 17%, 39% and 47% DM is too small for the error bars to be visible.) The point in red signifies a theoretical (0,0) representing a 0 MPa Young’s modulus for 0% DM PCLMA (since curing of solid structures to test under tensile loading is not possible). Cubic polynomial regression curve fitted (R^2^ = 0.99). (**C**) example stress–strain curves of each of the PCLMA samples.

PCLMA prepolymer of varying DM was photocured into tensile test-pieces (Figure 3A) and tested under tensile conditions. An increase in Young’s modulus was seen with increasing DM of the PCLMA material (Figure 3B). This can be explained as, increasing the number of methacrylate groups in the PCLMA allows for a greater degree of crosslinking in the cured product, resulting in a stiffer material. The result was an almost 30-fold increase in Young’s modulus from 0.12 ± 0.20 MPa to 3.51 ± 0.25 MPa (in the 17% and 77% DM PCLMA, respectively). The difference in Young’s modulus between each group of PCLMA was statistically significant (*p* ≤ 0.005), except for 1.5M20 vs 2M20 (39% and 47% DM, respectively) (one-way ANOVA with Tukey’s multiple comparisons). Example stress–strain curves for each of the different materials can be seen in Figure 3C. Due to the low DM of 0.5M20 and 0.75M20 PCLMA, photocuring these prepolymers did not result in sufficient crosslinking for the formation of bulk structures, so these samples were not included in the mechanical testing study.

### 2.3. Effect of Degree of Methacrylation on PCLMA Soluble Fraction

**Figure 4:**
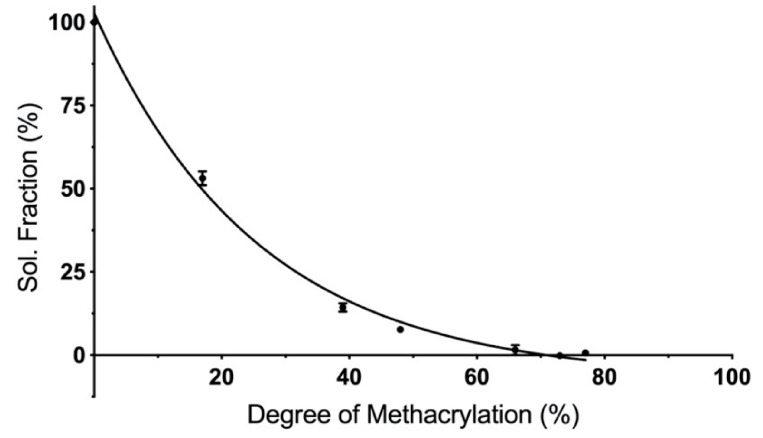
The influence of degree of methacrylation on the soluble fraction of cured PCLMA. Values displayed mean ± SD, N = 1, *n* = 3 (some SD values are too small for error bars to be visible).

Cured PCLMA of varying DM was solvated in dichloromethane (DCM) to remove any uncured prepolymer and to calculate the soluble fraction of the cured constructs. Increasing the DM of the PCLMA prepolymer led to a decrease in the soluble fraction (Figure 4). The least methacrylated PCLMA (1M20, 17% DM) had a soluble fraction of 53.1 ± 2.1%, but this dropped to 0.6 ± 0.8% as DM increased to 77% (2M95 PCLMA). The increased number of functional groups, in the higher DM PCLMA, allows for a greater percentage of prepolymer molecules to be incorporated into the cured polymer network. The 53% soluble fraction of the 1M20 PCLMA correlates well with the 17% DM. Here, each PCL triol molecule has an average of 0.51 functional groups so, statistically, around 50% of the molecules have one methacrylate group and are able to form a crosslinked network on photocuring. The remaining 50% are unmethacrylated and remain free to dissolve in the solvent.

### 2.4. Effect of Degree of Methacrylation on PCLMA Degradation

**Figure 5:**
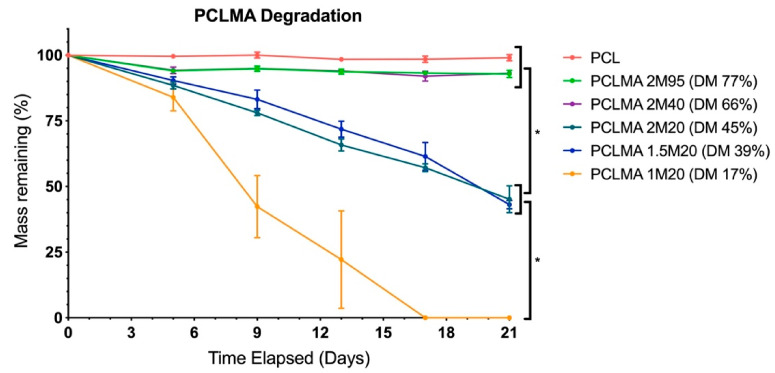
Accelerated degradation of PCLMA and PCL discs in 5M NaOH and the effect of degree of methacrylation. Value displayed mean ± SD, N = 1, *n* = 3. * denotes significant differences between groups at days 9, 13, 17 and 21 (two-way ANOVA with Tukey’s multiple comparisons).

Cured PCLMA of varying DM was subjected to an accelerated degradation study and compared with commercially available PCL. Figure 5 displays the percentage mass remaining at regular intervals over the course of the 3-week study. There was no significant degradation of the of the PCLMA with 77% DM (2M95) and 66% DM (2M40), with 92.9 ± 1.4% and 93.1 ± 0.2% remaining, respectively, after 21 days. This was comparable to the commercial PCL with 99.0 ± 1.1% still remaining after 21 days. The 45% DM (2M20) and 39% DM (1.5M20) PCLMA degraded faster than the previous 3 (45.1 ± 5.1% and 43.1 ± 1.6% remaining, respectively, after 21 days) but were not significantly different to each other at any time point. The 1M20 PCLMA degraded still faster with complete degradation occurring in 17 days. By day 9, the mass remaining of the PCL, 2M95 PCLMA and 2M40 PCLMA were significantly different to the 2M20 and 1.5M20 PCLMA (*p* < 0.05). Additionally, the mass remaining of the 1M20 PCLMA was significantly different to both the 2M20 and 1.5M20 PCLMA (*p* < 0.0001) (two-way ANOVA with Tukey’s multiple comparisons). These significant differences continued for all remaining time points (*p* < 0.0001 in both cases, at days 13, 17 and 21).

An increased DM of the PCLMA resulted in slower degradation rates, likely due to the increased crosslink density, meaning that more bonds need to be broken for material to degrade. These results are consistent with other studies in literature where an inverse relationship of polymer degradation rate and crosslink density have been shown with degradable poly(vinyl alcohol) scaffolds, PEG-co-PLA hydrogels and PCL-triol vs. PCL-diol acrylates [56,68,69]. Pashneh-Tala et al. also found that increasing the DM of PGS-methacrylate leads to a decreased degradation rate in enzymatic degradation with cholesterol esterase [13]. The large increases in degradation rate from the 2M95/2M40 PCLMA to the 2M20/1.5M20 PCLMA and to the 1M20 PCLMA is reflected by the large increases in soluble fractions of the same samples, seen in Figure 4. This could suggest that the removal of the soluble fraction leaves behind some microporosity that allows increased water permeation during the degradation process and therefore an increased degradation rate.

Like many synthetic polymers, PCL degrades by hydrolysis of the ester linkages in the polymer backbone [70,71]. Degradation of cured PCLMA and PCL in NaOH simulates accelerated hydrolysis due to the presence of OH^−^ ions which catalyse the hydrolysis of ester bonds [72]. The linear decrease in mass seen in the results is suggestive of surface degradation which is usually preferable for tissue engineering scaffolds as mechanical properties are maintained throughout the degradation process, unlike with bulk degradation [73].

The tuneable degradation rate of our PCLMA is of importance for tissue engineering applications where the scaffold degradation time should be matched to the time required for tissue regeneration. The increased degradation rate compared to linear PCL is also of interest for certain applications as linear PCL has been shown to remain in vivo for over three years [74] and long-term placement of materials within the body may lead to a chronic foreign body reaction and fibrous encapsulation [75]. For example, in the case of nerve regeneration, long-term placement of nerve guidance conduits has been shown to lead to pain and local discomfort due to compression of the regenerated nerve [76,77] so faster degradation times are desired.

### 2.5. Tunability of Photocurable Resins and Comparison of Our PCLMA with Other Materials

We have produced a tuneable, photocurable PCLMA material and demonstrated the ability to alter mechanical properties and degradation rate by varying the prepolymer DM. Other methods of influencing the mechanical properties and degradation rates of photocurable polymers have been reported in the literature; however, these methods, and the materials produced, have a number of drawbacks. Incorporating a solvent (non-reactive diluent) into the prepolymer resin has been shown to affect the mechanical properties of the cured resin. Lee et al. demonstrated a reduction of compressive moduli with increasing solvent content in a poly(propylene fumarate) resin [29]. Crosslinkers (reactive diluents) e.g., *N*-vinyl-2-pyrrolidone (NVP) may also be added to alter the crosslinking density. Jansen et al. demonstrated an increase in Young’s modulus (1.5–2.1 GPa) with increasing NVP content in a photocurable poly(d,l-lactide) resin [78]. Additives such as these may cause problems, such as the shrinkage of printed parts upon the extraction of solvents [29,37,38], resulting in significant distortions or dimensional changes [30]. Crosslinkers may also alter the properties of the cured material; for example, the hydrophilic nature of NVP can lead to an increased water uptake, resulting in swelling and reduction of mechanical properties [33,78]. 

To alter crosslinking density without the use of resin additives, the molecular structure of the prepolymer molecule may be changed. Increasing the molecular weight of a prepolymer molecule lengthens the chains between functional groups, so a looser network is formed with lower mechanical properties. This was demonstrated by Green et al., with a 75% increase in Young’s modulus of 300 g/mol PCL-triol acrylate compared to 900 g/mol prepolymer (6.9 vs 4.0 MPa) [56]. Elomaa similarly demonstrated an increase in PCLMA Young’s modulus (6.7 to 15.4 MPa) when decreasing prepolymer molecular weight from 1500 to 6000 g/mol [55].

As described in this study, varying the DM of a prepolymer is an alternative way to alter the crosslinking density of a cured material. By changing the number of functional groups able to facilitate crosslinking, the resultant properties of the cured material may be changed without the need for resin additives or different molecular weights. The practice of varying prepolymer DM to achieve a range of polymer properties has been demonstrated with a wide range of polymers including alginate, chondroitin sulphate, gelatin, hyaluronic acid and poly(glycerol sebacate) [13,24,25,26,27,28]. Jeon et al. demonstrated an increase in elastic modulus of methacrylated alginate from 43–175 kPa with DM increase of 4–25%, as well as a reduced rate of hydrolytic degradation [28]. Similar results have been shown with a gelatin-methacrylate polymer, with an increased in DM from 15% to 90% resulting in a Young’s modulus increase from 0.040–0.222 MPa and decreased enzymatic degradation [79]. Pashneh-Tala et al. also demonstrated that a change in DM of poly(glycerol sebacate) from 30–80% resulted in a 15-fold increase in Young’s modulus, from around 0.5 to 7 MPa, and a slower enzymatic degradation [13].

To the authors’ knowledge, the work presented in this study is the first to demonstrate PCL with varying degrees of methacrylation. Previous studies with PCL-triol-methacrylate or similar present products with only a single DM [55,56]. By fully characterising and varying the DM of the PCLMA in this study, a range of properties were seen for the cured polymer. The DM of PCLMA was varied from 17–77%, altering the number of functional groups available for crosslinking between the three-armed PCL molecules, and the subsequent crosslink density. By varying DM, the 3-arm PCL was functionalised with, on average, 0.5 (17% DM) to 2.3 (77% DM) methacrylate groups per molecule. Since the methacrylation reaction is a statistical process, this produces blends of mono-, di- and tri-methacrylated PCL molecules, with their relative ratios dependent on the DM. The advantage of this is that it allows us to fine-tune the mechanical properties and produce cured PCLMA with Young’s modulus anywhere in the range of 0.12–3.51 MPa (a 30-fold change). Additionally, this range of Young’s modulus is lower and wider than that by Green et al. (4.0–6.9 MPa) [56] and Elomaa at al. (6.7–15.4 MPa) [55] achieved by varying the molecular weight of their PCL-based prepolymers. This highlights the advantage of our method, being able to produce a more elastic material with a wider range of properties. The Young’s modulus of the photocurable PCLMA produced in this study is much lower than that of conventional PCL. Furthermore, the Young’s modulus of our PCLMA is lower than other reported photocurable materials, such as poly(propylene fumarate) (1.29 GPa [80]) and the various photocurable poly(d,l-lactide)-based materials mentioned previously (Young’s modulus ranging from 1.5–3.3 GPa [37,78]), yet higher than the Young’s modulus of photocurable hydrogel materials such as gelatin methacrylate (0.040–0.222 MPa [79]). This makes the material more suitable for use in tissue engineering for soft tissue applications, such as nerve guidance conduits, where the Young’s modulus of nerve tissue has been calculated to be around 0.6–8 MPa [81,82].

### 2.6. Cytocompatibility Testing of PCLMA

**Figure 6:**
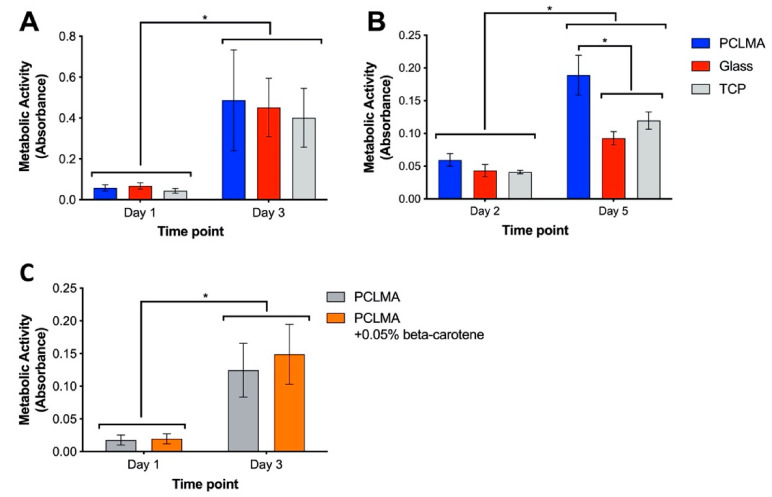
Cytocompatibility testing of 2M20 PCLMA, assessed by MTT assay. (**A**) cytocompatibility assessed with RN22 Schwann cells. (N = 2, *n* = 3). (**B**) cytocompatibility assessed with rat primary Schwann cells (N = 1, *n* = 3); (**C**) cytocompatibility testing of β-carotene-containing PCLMA, assessed by MTT assay (N = 3, *n* = 3). Values displayed mean ± SD. * denotes a significant difference (2-way ANOVA with Tukey’s multiple comparison).

Although PCL is widely reported as a biocompatible material, it was important to assess the functionalised PCLMA due to the addition of methacrylate groups which may result in changes to the body’s response to the material. It is especially important to assess the material’s cytocompatibility with tissue-specific cell types, ensuring the suitability of a material for specific applications. In our research, we are particularly interested in nerve guidance conduits and so the material was tested with a Schwann cell line (RN22) and rat primary Schwann cells. Cytocompatibility testing of 2M20 PCLMA was carried out via MTT assay. Metabolic activity of RN22 cells was compared on PCLMA-coated coverslips, plain glass coverslips, and tissue culture plastic (TCP) wells (Figure 6A). For each of the three sample types, metabolic activity significantly increased from day 1 to day 3, suggesting proliferation of cells between the two time points (*p* > 0.001, 2-way ANOVA with Tukey’s multiple comparison). There was no significant difference between the three groups at either day 1 or day 3, suggesting a comparable level of cytocompatibility. This experiment was repeated with primary Schwann cells obtained from rat sciatic nerve [83] (N = 1, *n* = 3) and a similar trend observed, with significantly increased metabolic activity on PCLMA, glass and TCP on day 5 vs. day 2 (Figure 6B). The increased metabolic on PCLMA vs. glass and TCP suggests that the PCLMA may exhibit a higher cytocompatibility with Primary Schwann cells compared to the controls; however, it should be noted that increased metabolic activity does not necessarily indicate an increased cell number.

β-carotene was included in the PCLMA resin to act as a photoabsorber during the production of 3D structures via microstereolithography. The cytocompatibility of β-carotene-containing PCLMA was therefore tested by performing an MTT assay with RN22 Schwann cells on discs of PCLMA and PCLMA with 0.05% β-carotene (Figure 6C). This was to replicate the bulk structures that may be implanted in vivo in the form of a tissue engineering scaffold. No significant difference was found between the PCLMA and PCLMA with β-carotene at either time point. This suggests that the β-carotene had no adverse effect on the viability of the RN22 cells. The increase in metabolic activity from day 1 to day 3 was significant for both materials (*p* < 0.0001, 2-way ANOVA with Tukey’s multiple comparison) suggesting a large amount of proliferation on both samples and indicating their common cytocompatibility.

The cytocompatibility of this PCLMA resin could be an improvement on other photocurable forms of PCL which have shown lower cytocompatibility compared to TCP (e.g., mesenchymal stem cells cultured on vinyl-terminated PCL [33] and fibroblasts cultured on PCLMA [55]). A previous study from our group also highlighted the cytocompatibility of similar PCLMA material to that reported in this study. Neuronal and Schwann cells were cultured on PCLMA and shown to exhibit comparable viability compared to TCP controls as assessed via a live/dead assay [84]. In the same study, porous PCLMA nerve guidance conduits were used to repair rat sciatic nerve injuries and resulted in good tissue integration.

### 2.7. Production of 3D Structures via Microstereolithography

**Figure 7:**
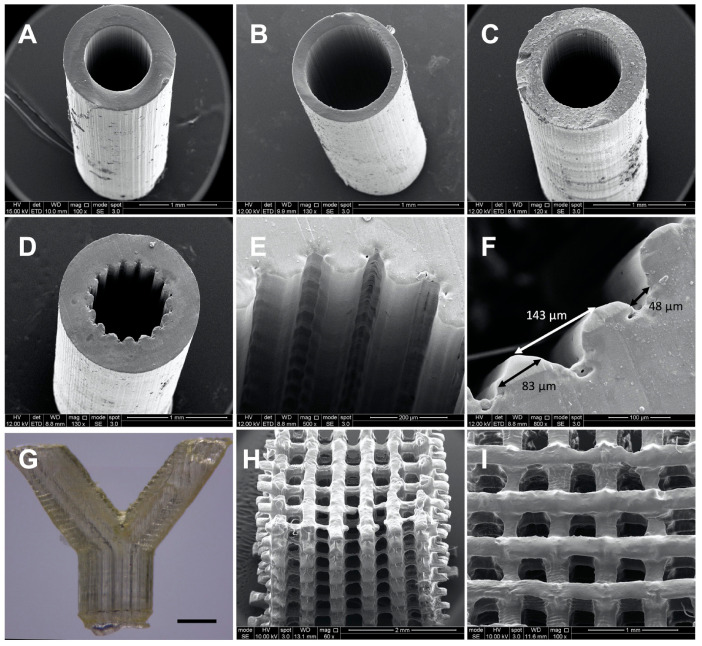
SEM images (**A**–**F**, **H**,**I**) and optical microscopy image (**G**) of constructs produced by microstereolithography using 2M20 PCLMA resin. (**A**–**C**) simple tubes produced using PCLMA resin containing no photoabsorber (**A**,**B**) and containing 0.075% β-carotene (**C**). (**D**–**I**) more complex 3D structures produced using PCLMA resin containing β-carotene as a photoabsorber. (**D**/**E**) grooved tube, (**F**) annotated image of a grooved tube showing the average groove width (48 µm), strut width (83 µm) and peak-to-peak width (143 µm); (**G**) bifurcated tube, scale bar: 1 mm; (**H**/**I**) woodpile structure (side view and top view, respectively).

2M20 PCLMA resin (47% DM) was used on an in-house built microstereolithography set-up to produce 3D structures of varying designs. Simple constructs with minimal features and a constant cross section were possible to create with PCLMA resin containing no photoabsorber. Simple tubes, both thin-walled and thick-walled designs (Figure 7A,B), were produced of high quality, with internal diameters of 880 µm and 920 µm, respectively, and wall thicknesses 290 µm and 150 µm, respectively. For comparison, Figure 7C shows a simple tube produced with PCLMA resin containing 0.075% β-carotene (900 µm internal diameter, 280 µm wall thickness) with comparable quality to the tubes in Figure 7A,B. Both types of tube had straight sides, a circular internal diameter, and reproducible wall thicknesses. This was achieved by carefully balancing the laser power and speed at which the z-stage descended, and by heating the resin in a 70 °C water bath during the printing process.

When curing parameters were not optimised (i.e., high laser power, slow stage speed or when the resin was used at room temperature), lateral ridges were seen in the cured construct (Figure S1). This effect was due to over-curing of the available resin at the surface of the vial as the stage descended. Additionally, the ridges formed as the higher viscosity of the room-temperature resin prevented the flow of the resin over the descending construct and disrupted the continual curing process.

**Figure 8:**
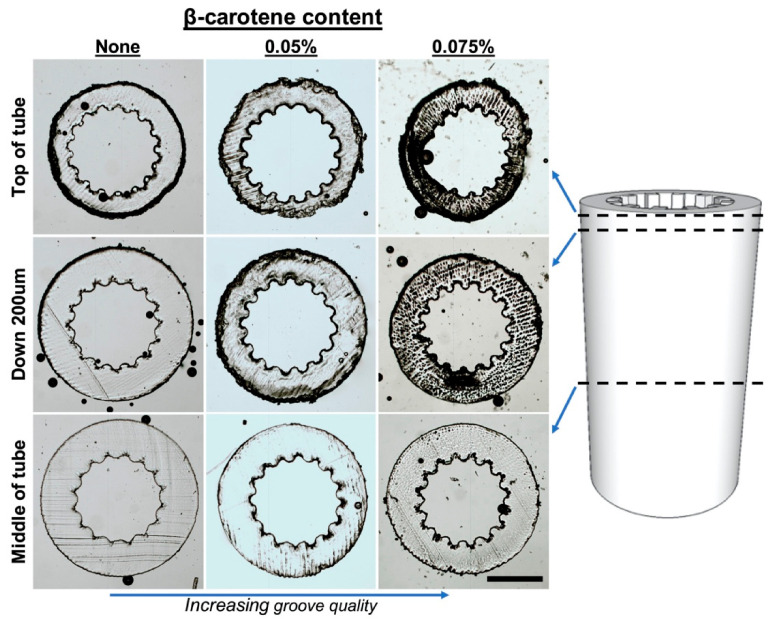
Demonstration of laser depth penetration control, and increased microstereolithography scaffold quality, with the addition of β-carotene in the PCLMA resin. Sections of grooved tubes (obtained using a cryostat) showing the appearance of the grooves down the length of the tubes. Groove quality decreases towards the middle of the tube but increases with an increasing amount of β-carotene in PCLMA resin. Scale bar: 500 µm.

For the production of complex 3D structures with small features or non-constant cross sections, β-carotene was incorporated into the PCLMA resin to act as a photoabsorber. Tubes were produced with a grooved pattern along the luminal surface for the application of nerve guidance conduits (Figure 7D–F). To obtain a good resolution, the addition of a photoabsorber was necessary. Without a photoabsorber, the laser light was able to penetrate deeper into the vial of prepolymer resin and the grooves further down the construct started to merge due to over-curing (see Figure 8). β-carotene was selected as a photoabsorber due to its solubility in our PCLMA resin and its suitable absorption spectrum. It has a large absorption in the violet-blue-green range, with absorption maxima of 449, 478, and 518 nm, and absorbs well at 405 nm, a wavelength commonly used in commercial stereolithography set-ups. It was found that concentrations of 0.05–0.075% β-carotene seemed to be the optimum level of photoabsorber for the production of 3D structures using the custom-built stereolithography set-up. When too much photoabsorber is present, features become thin and fragile due to under-curing. This effect was observed when using high concentrations of β-carotene in PCLMA resins, resulting in under-cured PCLMA tubes (Figure S2).

With the inclusion of 0.075% β-carotene, the grooved structures were reproduced with a good definition. The grooved features had a 143 ± 14 µm peak to peak distance, measured 83 ± 10 µm across the struts and measured 48 ± 10 µm across the grooves. A bifurcated tube (Figure 7G) was produced using PCLMA resin containing 0.05% β-carotene. The low amount of β-carotene meant that the transparent nature of PCLMA was maintained. This makes it possible to visualise an open lumen throughout the tube, indicating a good control over cure depth. Woodpile structures (strut size 217 ± 39 µm) were also produced using PCLMA resin containing 0.075% β-carotene (Figure 7H,I). The good control over cure depth allowed the alternating struts to be distinct and evenly spaced.

This study has demonstrated the suitability of β-carotene as a photoabsorber in µSL in enabling the production of high-quality 3D structures, as well as its cytocompatibility when incorporated into cured PCLMA. It should be noted that the stability of β-carotene is affected by temperature and degradation of β-carotene has been shown during storage at different temperatures. For this reason, heating times should be minimised where possible and temperature should be considered during long-term storage.

## 3. Materials and Methods

All chemicals and reagents used were purchased from Sigma-Aldrich, Dorset, UK, unless otherwise stated.

### 3.1. PCL Methacrylation

**Figure 9:**
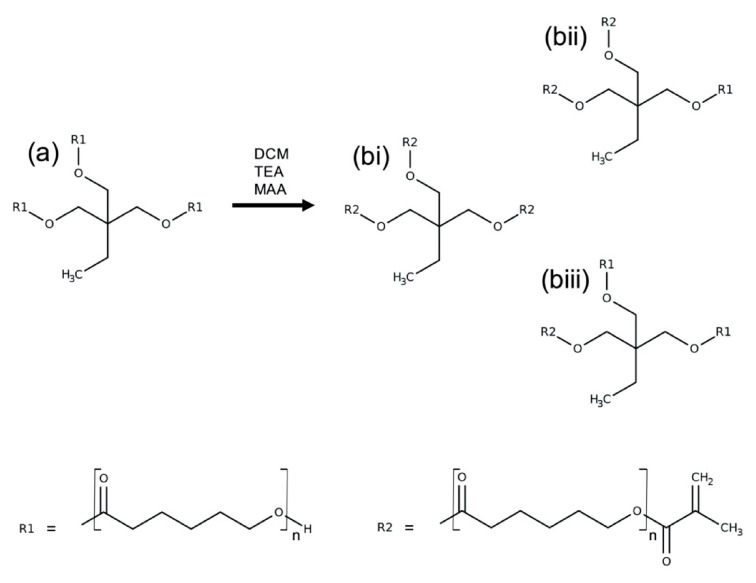
PCL methacrylation reaction scheme. PCL triol (**a**) dissolved in DCM, was reacted with MAA in the presence of TEA to produce three-armed PCLMA (**b**). Depending on the reaction time and the reactant concentrations, the PCL triol could become fully methacrylated, with all three arms expressing a methacrylate group (**bi**) or partially methacrylated (**bii**,**biii**).

PCL triol (M_n_: 900 g/mol) was reacted with methacrylic anhydride (MAA) in the presence of an equimolar amount of triethylamine (TEA) to produce photocurable poly(caprolactone-methacrylate) (PCLMA) (see Figure 9 for reaction scheme). PCL triol was dissolved in DCM (Fisher Scientific, Loughborough, UK) at a ratio of 1:10 (*w*/*v*) along with TEA. The reagents were cooled to 0 °C, purged with nitrogen and the MAA was added dropwise. The reaction mixture was allowed to gradually come up to room temperature and the reaction was continued for the predetermined time. The resulting PCLMA solution was washed three times with 1 M hydrochloric acid (Fisher Scientific), followed by two washes with deionised water. The solvent was then removed from the PCLMA by rotary evaporation. The PCLMA was washed in methanol and precipitated at −80 °C (repeated 3–4 times) followed by further solvent removal by rotary evaporation.

For 1 mol of PCL triol, 3 mol of hydroxyl groups are available for functionalisation. A 1:1 (PCL:MAA) molar ratio reaction is therefore described as 1 molar equivalent of PCL triol reacting with 3 molar equivalents of MAA and TEA. In order to vary the degree of methacrylation, the reaction was carried out with varying molar ratios in the range of 1:0.5 to 1:2 (representing 1.5 and 6 mol of MAA per mol of PCL triol, respectively—a 0.5× molar insufficiency to a 2× molar excess). To further vary the degree of methacrylation, the reaction time was varied from 20 to 95 h. Table 1 outlines the different combinations of molar excess/reaction times investigated and explains the notation used to describe the different reactions. i.e., 2M20 refers to a 1:2 reaction with a 20 h duration.

### 3.2. Nuclear Magnetic Resonance (NMR) Spectroscopy

Proton nuclear magnetic resonance spectroscopy (Bruker AV3HD-400 NMR spectrometer, Birrica, MA, USA) was performed to characterise the PCL triol and PCLMA and allow calculation of the degree of methacrylation. 20 mg samples of polymer were solvated with 0.5 mL of deuterated chloroform and analysed at 400 Hz at room temperature. Spectra peaks were referenced to CDCl_3_ at 7.26 ppm and identified with the help of a previous publication by Elomaa et al. [55] (see Figure 1). For quantitative analysis of degree of methacrylation, the integrals of peaks representing methacrylate protons at 6.09 ppm and 5.55 ppm (i, j) and the methylene group adjacent to the methacrylate group at 4.14 ppm (a′′) were integrated, relative to integral of the methyl group at 0.88 ppm (h). Analysis was performed using Mnova (Mestrelab).

### 3.3. Photocurable Resin Preparation and Photocuring

Photocurable resin was prepared by mixing PCLMA prepolymer with 2% (*w*/*w*) photoinitiator (diphenyl(2,4,6-trimethylbenzoyl)phosphine oxide/2-hydroxy-2-methylpropiophenone, 50/50 blend) using a vortex mixer followed by 10 min sonication to remove air bubbles.

To increase the resolution of 3D printed structures, β-carotene was added to some resins to act as a photoabsorber. Here, β-carotene was added to the prepolymer at 0.05–0.075% (*w*/*w*) and stirred at 60 °C for 1 h before photoinitiator was added.

For mechanical testing, soluble fraction, degradation and cell culture studies, PCLMA resin was dispensed into silicone moulds and photocured using a 100 W UV lamp (Omnicure S1000 curing lamp) with a 10 min exposure time, flipping the moulds half way.

### 3.4. Mechanical Testing

The Young’s modulus of the PCLMA was calculated via tensile testing (EnduraTEC ELF 3200 load frame system, Bose, 450 N load cell). PCLMA resin was cured into tensile test pieces, adapted from ASTM D638 Type V specimen. Tensile testing was performed at a strain rate of 0.1 mm s^−1^ (initial grip separation, 22.5 mm) until fracture or slipping occurred. Young’s modulus was calculated from the linear portion of the resulting stress strain curves (*n* = 4–7).

### 3.5. Soluble Fraction

The soluble fractions of the cured PCLMA were measured to calculate the amount of PCLMA not incorporated into the crosslinked networks. PCLMA was cured into discs (4 mm diameter, 1.3 mm thickness). Samples were dried under vacuum, weighed and washed in DCM for 5 days to swell and remove any uncured prepolymer. The samples were then removed from the solvent, dried under vacuum and weighed again to calculate percentage mass loss (*n* = 3).

### 3.6. Degradation Study

An accelerated degradation study was performed on cured PCLMA discs to compare the relative degradation rate of the PCLMA with varying DM. Degradation rate was compared to commercially available, linear PCL (M_n_ 80,000 Da). PCLMA was cured into discs (4 mm diameter, 1.3 mm thickness) and the soluble fraction removed. The PCLMA discs and similar discs of PCL were dried under vac, weighed and placed in 5M NaOH on a rocker. Samples were removed at time points of 5–21 days, washed three times in PBS and dried under vacuum. Each disc was weighed and compared to its individual start weight to calculate the percentage weight remaining (*n* = 3).

### 3.7. Cytocompatibility Study

RN22 Schwann cells and rat primary Schwann cells were used in a cytocompatibility study of cured PCLMA resin. RN22 cells (European Collection of Authenticated Cell Cultures, ECACC 93011414) [85], a transformed cell line derived from rat Schwann cells, were maintained in culture using Dulbecco’s Modified Eagle Medium (DMEM) supplemented with 10% foetal calf serum (FCS), 1% l-glutamine, 1% penicillin/streptomycin and 0.25% amphotericin B. Cells were cultured in a humidified atmosphere at 37 °C, 5% CO_2_. Primary Schwann cells were obtained from rat sciatic nerve using methods described previously [83] and maintained as above.

For cytocompatibility testing of cured PCLMA, glass coverslips (22 mm diameter) were functionalised by treatment with 3:1 solution of sulphuric acid:hydrogen peroxide (60 min), followed by treatment with 10% 3-(Trimethoxysilyl)propyl methacrylate (MAPTMS) in toluene (24 h). Coverslips were spin-coated (Laurell Technologies WS-400B-6NPP/Lite) with 150 µL of PCLMA resin (3000× *g* rpm, 30 s) and cured UV cured (Omnicure S1000 curing lamp) for 100 s. The coated coverslips were then washed in isopropanol (IPA) for 1 week prior to cell culture, changing the IPA at least twice in this time. Washing cured polymer in IPA partially removed the soluble fraction however some remained due to the lower solubility of the polymer in IPA compared to in DCM. PCLMA-coated coverslips and plain glass coverslips were placed in 12-well plates, sterilised with 70% ethanol for two hours and washed 3 times with PBS. Confluent RN22 cells were trypsinised and seeded onto the coverslips as well as empty TCP wells (25,000 cells in 3 mL media). Cultures were maintained for either 1 or 3 days before an MTT assay was performed. This experiment was repeated with primary Schwann cells and cultures were maintained for 2 to 5 days before performing the MTT assay. The longer duration of the primary Schwann cell culture time points was chosen due to their comparatively slower growth compared to the RN22 cells.

PCLMA resin containing β-carotene was cured into discs and tested for cytocompatibility against plain PCLMA discs containing no β-carotene. PCLMA resins were prepared with 0% and 0.05% β-carotene. Polymer discs (12 mm diameter, 2 mm thickness) were cured in PDMS wells and washed for one week in IPA prior to cell culture, changing the IPA at least twice in this time. The PCLMA discs were placed in 12-well plates, sterilised with 70% ethanol for two hours and washed 3 times with PBS. Confluent RN22 cells were trypsinised and seeded into each wells (48,000 cells in 3 mL media). After 24 h, discs were transferred to new wells with fresh medium and cultures were maintained for either 1 or 3 days from the time of seeding before an MTT assay was performed. 

### 3.8. MTT Assay

At the termination of the cultures, medium was removed, and wells were washed once with PBS. MTT solution (0.5 mg/mL in PBS, filter sterilized) was added to each well and samples incubated for 1 hour at 37 °C. For coverslip cultures, 1 mL MTT solution was used and, for polymer disc cultures, 2.5 mL MTT solution was used. The MTT solution was removed, and the stain was eluted with 350 µL acidified IPA (12.5 mM HCl in IPA). 100 µL duplicate samples of the eluted stain were extracted from each well, pipetted into a 96-well plate and read on an absorbance plate reader (Bio-TEek ELx 800) at 540 nm (reference 630 nm). Assays were performed in triplicate (*n* = 3) and repeated up to 3 times (N = 3).

### 3.9. Microstereolithography

**Figure 10:**
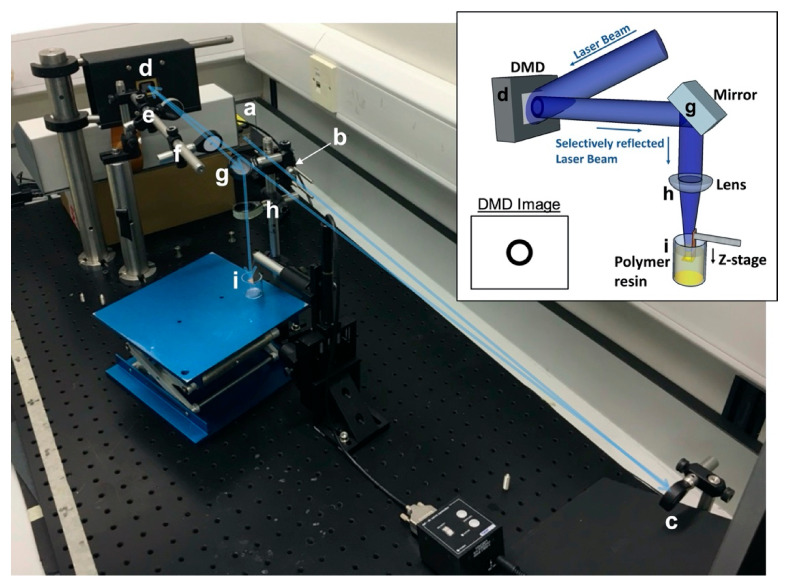
The microstereolithography (µSL) set-up. Inset: Schematic of the set-up. An expanded laser beam hits the DMD which selectively reflects the laser beam in the shape of the desired image which is focused on the surface of a vial of prepolymer resin. Example DMD image shown for use in tube production. Main image: The experimental µSL set-up. A laser beam from source (**a**) is passed through a spatial filter (**b**) and reflected off a mirror (**c**) towards a DMD (**d**). The selectively reflected image from the DMD is passed through a 25 cm focal length lens (**e**) followed by an iris (**f**) to remove multiple images caused by diffraction. The image is then reflected down by a second mirror (**g**) and focused by a 10 cm focal length lens (**h**) onto the stage in the vial of prepolymer resin (**i**). The blue arrow illustrates the laser beam path.

PCLMA resin was used on an in-house built, top-down microstereolithography set-up (modified from that described by Pateman et al. [5]) to produce 3D structures (Figure 10). Briefly, a 405 nm violet laser (Vortran Stradus, Vortran Laser Technology Inc., Roseville, CA, USA) was passed through a series of beam-expanding optics to reflect off a digital micromirror device (DMD) (DLP^®^ 0.7” XGA, Texas Instruments Inc., Dallas, TX, USA). The reflected image (scaffold cross-section) was passed through a set of focussing optics and focused on the platform of a one-axis motorised translation stage (Thorlabs Ltd., Newton, NJ, USA). The stage was controlled by computer software (APT Software 1.8.2.2, Thorlabs Ltd.) allowing precisely controllable, micrometre adjustment in the z-direction. A vial of photocurable prepolymer resin was placed under the stage with the level of the resin matching the level of the stage at the focal point of the projected image.

Cross-sectional images of the designed scaffold were uploaded onto the DMD so that, when the laser was turned on, the cross-sectional image was projected onto the stage at the surface of the prepolymer resin, curing the first part of the structure and attaching it to the stage. The stage was set to descend and the prepolymer continually cured at the surface while the surrounding resin flowed over the cured structure to refresh the surface resin. Laser powers of 10–40 mW were used, with stage speeds of 0.03–0.005 mms^−1^. To decrease the viscosity of the prepolymer resin and allow the resin to easily flow over the descending structure, the vial of prepolymer was placed in a 70 °C water bath during the printing process. Heating the resin has previously shown to be beneficial in reducing resin viscosity and allowing faster print times [30,86].

**Figure 11:**
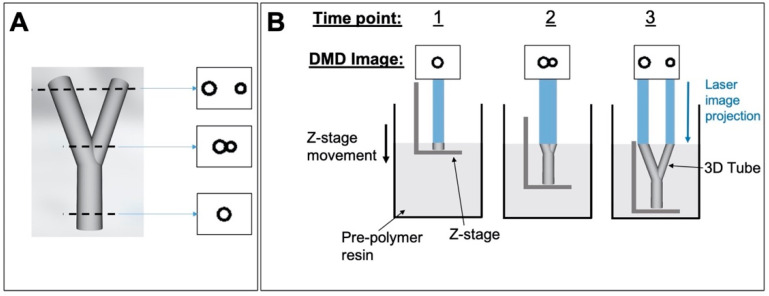
Production of 3D structures via microstereolithography. (**A**) 3D bifurcated tube designed in SolidWorks and three example cross-sectional images obtained from the ‘slicing’ process; (**B**) the process of curing a 3D tube by sequentially displaying the cross-sectional images on the DMD (three arbitrary time points shown.).

For simple structures with a constant cross-section (i.e., simple tubes), the DMD image remained constant throughout the curing process. For more complex 3D scaffolds (i.e., bifurcated tubes and woodpile scaffolds), multiple DMD images were displayed in sequence as the stage descended, to correspond with the shape of the current cross-section being cured (Figure 11). The layer thickness of each cross section was determined by the speed of the stage and the time that each image was displayed for.

Structures produced by microstereolithography washed in IPA to remove uncured polymer. For imaging by scanning electron microscopy (SEM), samples were air dried, sputter coated with gold (SC500, Emscope) and imaged with an FEI Inspect F50 scanning electron microscope.

### 3.10. Statistical Analysis

Statistical analysis was performed with GraphPad Prism, San Diego, CA, USA. To test for significant differences between groups, ANOVA was used, followed by Tukey’s multiple comparison tests. For data affected by one factor, one-way ANOVA was used and for data affected by two factors, two-way ANOVA was used. *p* < 0.05 was used to determine a statistical difference. All data is presented as mean ± standard deviation (SD). Sample sizes are presented as N = experimental repeats, *n* = replicates per experiment i.e., N = 1, *n* = 3 signifies an experiment performed once in triplicate.

## 4. Conclusions

PCL triol was methacrylated to varying degrees by varying the reaction parameters in the polymer synthesis reaction. The result was PCLMA prepolymer which mixed with a photoinitiator to produce a photocurable prepolymer resin. The cured PCLMA was shown to be cytocompatible, supporting the growth of both primary and RN22 Schwann cells. The PCLMA resin was successfully used in a custom microstereolithography set-up to produce a variety of structures. Simple tubes were produced with plain PCLMA resin, and the inclusion of β-carotene as a photoabsorber allowed the production of more complex structures including bifurcated tubes and woodpile scaffolds. β-carotene was also demonstrated as a cytocompatible photoabsorber. By varying the DM of the PCLMA from 17–77%, the mechanical properties of the cured material could be tuned, with achievable Young’s modulus in the range of 0.12–3.51 MPa, with also significant changes in the degradation rate. This allows for the use of this material in a variety of applications in scaffold production for tissue engineering. Future studies could investigate the cytocompatibility of the different DM PCLMA materials as well as assessing the long-term biocompatibility to the material and its degradation products via in vivo studies.

## Figures and Tables

**Table 1:** Reaction parameters used to produce PCLMA and the resulting degree of methacrylation (DM). DM was altered by varying the molar ratio of reactants and the reaction time. N = 1, *n* = 1, except for 2M20 where N = 3, *n* = 1 so mean ± SD shown only for 2M20.

	Reaction Notation	Reaction Ratio (Mol of PCL: Molar Excess of MAA/TEA)	Moles of MAA/TEA per 1 Mole of PCL	Reaction Time	Resulting Degree of Methacrylation
**Varying reaction ratio**	0.5M20	1:0.5	1.5	20 h	8%
	0.75M20	1:0.75	2.25	20 h	11%
	1M20	1:1	3	20 h	17%
	1.5M20	1:1.5	4.5	20 h	39%
	2M20	1:2	6	20 h	47 ± 2%
**Varying reaction time**	2M20	1:2	6	20 h	47 ± 2%
	2M40	1:2	6	40 h	66%
	2M68	1:2	6	68 h	73%
	2M95	1:2	6	95 h	77%

## Data Availability

The data presented in this study are openly available on FigShare at DOI: 10.15131/shef.data.13551422.

## Supplementary Materials

The following are available online. Figure S1: Optical microscopy image of a PCLMA tube produced via microstereolithography with non-optimal curing conditions. Lateral ridges can be seen due to overcuring of the material as a result of high laser power and the higher viscosity of room temperature resin. Scale bar: 1 mm. Figure S2: The effect of light absorbers in µSL. Side view (A) and top view (B) of an under-cured tube produced by µSL with PCLMA containing too high a concentration of β-carotene (0.2%). Scale bar: 1 mm.

## Sample Availability

Samples of the compounds are not available from the authors.
