# Supplementary material for: A Tuneable, Photocurable, Poly(Caprolactone)-Based Resin for Tissue Engineering—Synthesis, Characterisation and Use in Stereolithography
Source: Molecules. 2021 Feb 24;26(5):1199. doi: 10.3390/molecules26051199 (PMC7956195; doi:10.3390/molecules26051199)
Supplement: Supplementary file 1 [file molecules-26-01199-s001.pdf]

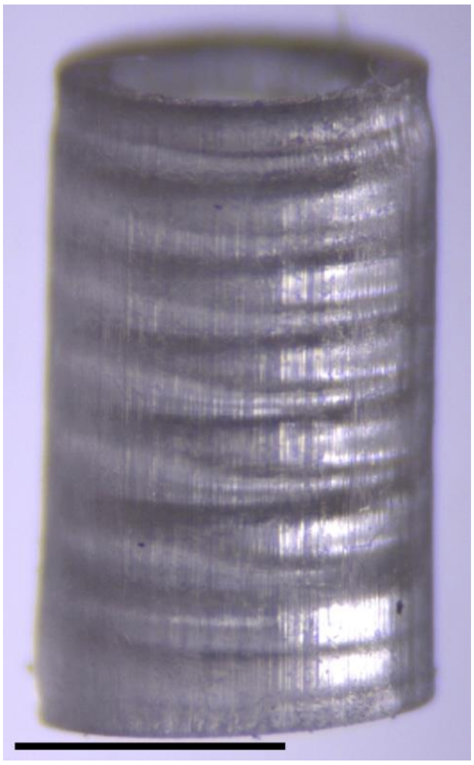

**Figure S1.** Optical microscopy image of a PCLMA tube produced via microstereolithography with non-optimal curing conditions. Lateral ridges can be seen due to overcuring of the material as a result of high laser power and the higher viscosity of room temperature resin. Scale bar: 1 mm.

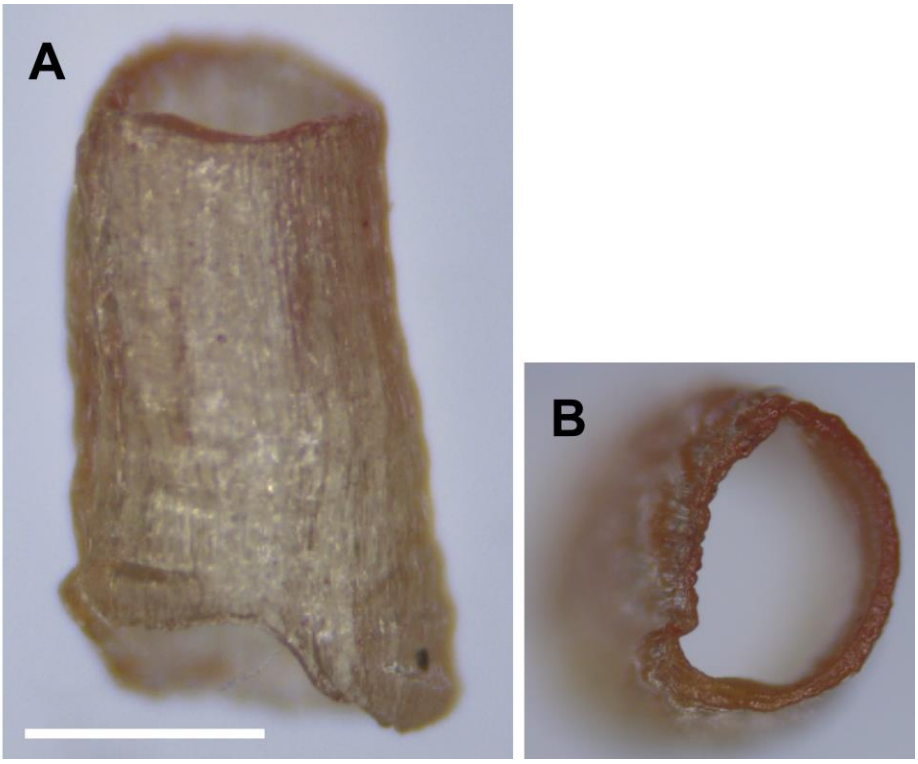


**Figure S2.** The effect of light absorbers in μSL. Side view (**A**) and top view (**B**) of an under-cured tube produced by μSL with PCLMA containing too high a concentration of β-carotene (0.2%). Scale bar: 1 mm.
